# Kirigami Engineering of Suspended Graphene Transducers

**DOI:** 10.1021/acs.nanolett.2c01256

**Published:** 2022-06-27

**Authors:** Chunhui Dai, Yoonsoo Rho, Khanh Pham, Brady McCormick, Brian W. Blankenship, Wenyu Zhao, Zuocheng Zhang, S. Matt Gilbert, Michael F. Crommie, Feng Wang, Costas P. Grigoropoulos, Alex Zettl

**Affiliations:** # Department of Physics, University of California, Berkeley, California 94720, United States; ‡ Materials Sciences Division, Lawrence Berkeley National Laboratory, Berkeley, California 94720, United States; § Kavli Energy NanoSciences Institute at the University of California Berkeley and the Lawrence Berkeley National Laboratory, Berkeley, California 94720, United States; ∥ Laser Thermal Laboratory, Department of Mechanical Engineering, University of California, Berkeley, California 94720, United States

**Keywords:** graphene kirigami, graphene NEMS, acoustic transducer

## Abstract

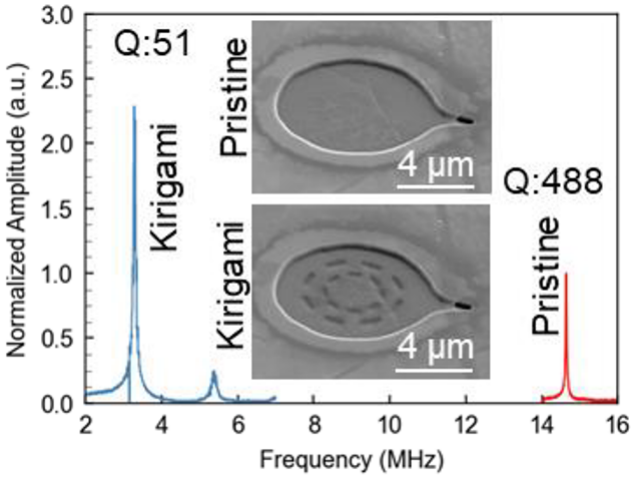

The low mass density and high mechanical strength of graphene make it an attractive candidate for suspended-membrane energy transducers. Typically, the membrane size dictates the operational frequency and bandwidth. However, in many cases it would be desirable to both lower the resonance frequency and increase the bandwidth, while maintaining overall membrane size. We employ focused ion beam milling or laser ablation to create kirigami-like modification of suspended pure-graphene membranes ranging in size from microns to millimeters. Kirigami engineering successfully reduces the resonant frequency, increases the displacement amplitude, and broadens the effective bandwidth of the transducer. Our results present a promising route to miniaturized wide-band energy transducers with enhanced operational parameter range and efficiency.

Suspended graphene is an attractive platform for energy transduction, for example in converting an electrical or optical signal into mechanical motion. Typically, the size (i.e., diameter) of the suspended membrane dictates the associated operational frequency, bandwidth, and displacement amplitude. For example, membranes of micron scale operate efficiently in the radio frequency range,^1−10^ while membranes of millimeter or centimeter scale operate efficiently in the acoustic to ultrasonic range.^11,12^

For certain applications, it would be highly desirable to alter the operating frequency, bandwidth, and displacement amplitude of the transducer, while maintaining the gross geometrical footprint of the suspended membrane. For example, a small-size membrane operating at low frequencies with reasonable bandwidth and displacement amplitude has implications for ultrasonic and audio transduction. Previous studies have shown that electric field gating^13^ and photodoping^14^ can tune the resonant frequency of a graphene mechanical transducer, but these methods are more effective in upshifting, rather than downshifting, the resonant frequency. Furthermore, the bandwidth of a graphene transducer normally shrinks (quality factor, *Q*, increases) when the resonant frequency is decreased because of reduced energy dissipation,^15^ and the displacement amplitude (*d*) of the vibrating membrane also decreases with the reduced membrane radius (*r*) as^16,17^
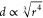
1

Previous studies^6,18^ have demonstrated methods to increase *Q* by engineering the graphene geometry, but none have shown how to effectively decrease *Q*.

Kirigami engineering is a promising alternative to precisely tailor the mechanical properties of suspended graphene membranes.^19−21^ Theoretical studies have shown that kirigami patterns can effectively alter graphene’s stiffness and significantly reduce its spring constant,^20,21^ which in principle permit concomitant tuning of the resonant frequency, bandwidth, and displacement amplitude. Graphene kirigami structures have been created through the lithography process in an aqueous environment and have been actuated by various static forces.^19^ Another approach has been to employ kirigami design to a graphene/polymer bilayer.^22,23^ However, it remains a challenge to realize and actuate graphene kirigami patterns outside an aqueous environment or without a supporting adlayer. In addition, past studies of graphene kirigami have focused only on static or very low frequency forces.^19,20^ Higher frequency response of graphene kirigami structures remain largely unexplored.

In this Letter, we demonstrate suspended graphene kirigami transducer structures ranging in size from a few micrometers to a few millimeters, operated primarily in vacuum. We find that appropriate kirigami engineering effectively lowers the operational frequency, decreases the *Q*, and improves the displacement amplitude of the transducers.

The kirigami “cuts” are created in preformed, suspended graphene (CVD grown, monolayer, or multilayer) using either focused ion beam milling (using helium or gallium ions) or pulsed laser ablation (using a femtosecond laser) (see SI for details). Figure 1a and b shows schematically the cutting process for the ion milling and laser ablation, respectively. We fabricate two types of kirigami patterns, which we term “spiral” and “circular”, both of which significantly alter the mechanical compliance (effective spring constant) of the membrane at its periphery but leave the central portion of the membrane intact. Figure 1c–f shows examples of spiral and circular kirigami-engineered suspended graphene membranes at three different characteristic size scales: panels c and d show, respectively, circular and spiral cut monolayer graphene with a diameter of 8 μm; panel e shows spiral cut trilayer graphene with a diameter of 30 μm, and panel f shows spiral cut multilayer graphene (∼100 nm thick) with a diameter of 8 mm. Both the spiral and circular kirigami patterns transform the original circular mechanically homogeneous diaphragm into a uniform central membrane supported by eight peripheral soft flexures. Thus, the deformation mechanics of the membrane are not typically dominated by any preformed tension of the membrane but by the bending-stiffness of the flexures. When a force is applied normal to the membrane, the major deformation occurs at the flexure and the central plane experiences minimum geometry change. Hence, both the effective spring constant and built-in stress of the graphene membrane are significantly modified because of the kirigami cuts.

**Figure 1:**
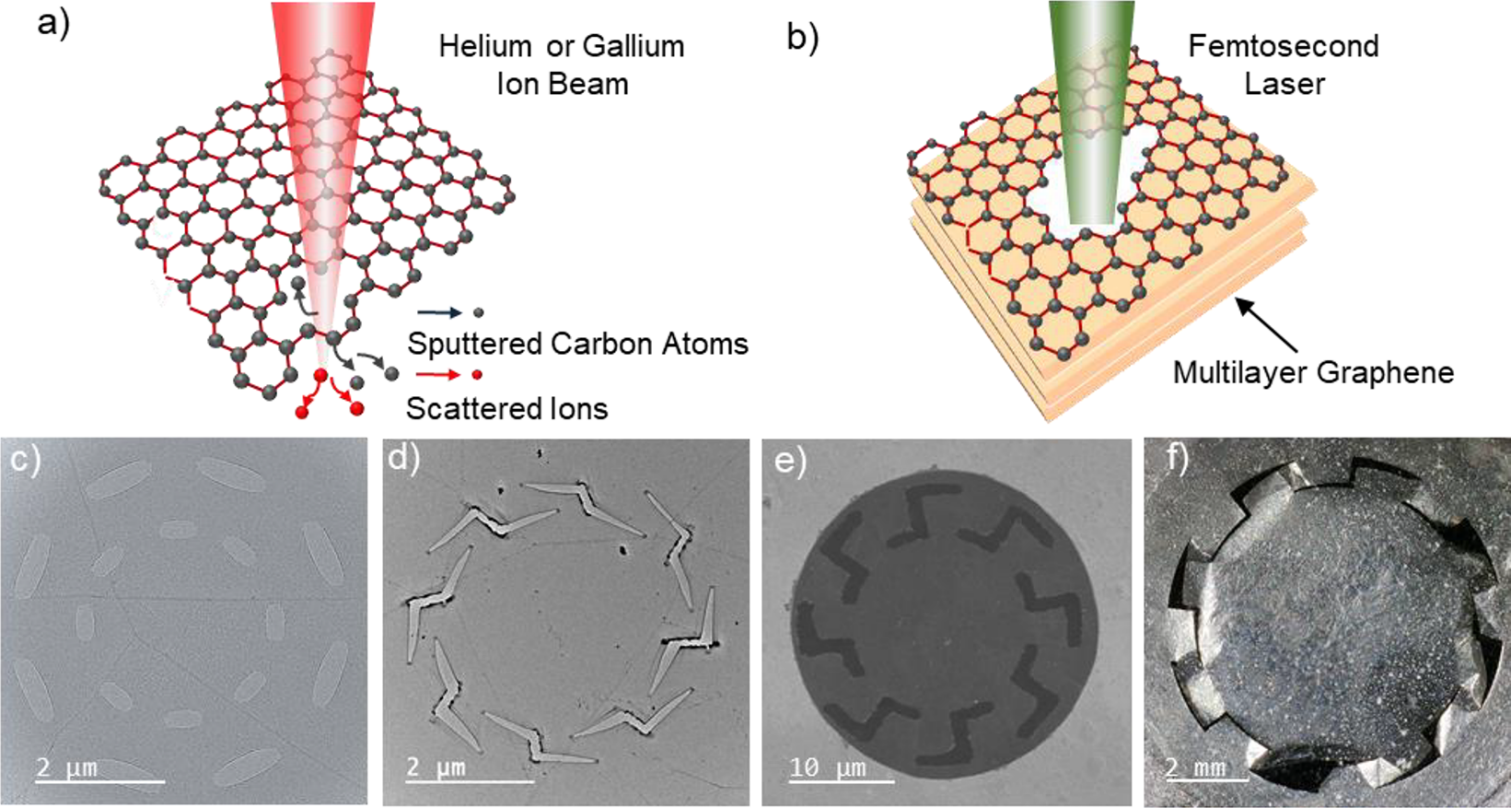
Schematics and images showing the fabrication of graphene kirigami patterns. (a) Schematics of monolayer graphene kirigami patterning process using helium or gallium ion beam milling. (b) Schematics of multilayer graphene kirigami patterning process using laser ablation. (c–f) Kirigami patterns with different dimensions are demonstrated on suspended graphene membranes: (c) TEM image of a circular kirigami pattern defined on a monolayer graphene using a gallium ion beam, (d) TEM image of a spiral kirigami pattern defined on a monolayer graphene using a helium ion beam, (e) SEM image of a spiral kirigami pattern defined on a trilayer graphene using laser ablation, and (f) optical image of a spiral kirgami pattern defined on a ∼100 nm thick many-layer graphene thin film using laser ablation.

For detailed transduction characterization, we fabricate the suspended graphene membranes, with kirigami cuts, on a micromachined silicon/silicon oxide wafer that allows consistent mounting and actuation in a vacuum or low pressure air environment (see SI for details). Figure 2a shows schematically the cross-section of the membrane mount. To characterize the response of the kirigami-engineered graphene transducers, we drive the membrane with a frequency-modulated optical field (laser) and measure the out-of-plane displacement response with an independent optical Fabry–Perot interferometer^24−27^ (see SI for details). This technique is best suited to the smaller-diameter (few microns) membranes; hence, in the presentation of data and discussion below, we concentrate on transducers of this size scale. However, our findings and analysis should be applicable to the larger transducers as well, including those of millimeter or even centimeter scale. We also note that membranes cut with radical spiral kirigami patterns can be susceptible to bending or folding of the graphene at the outside corners of the cut, especially after sustained heavy actuation (Figures 1f and S3). Therefore, for consistency of results, we limit our detailed experimental characterization to circular kirigami patterns.

**Figure 2:**
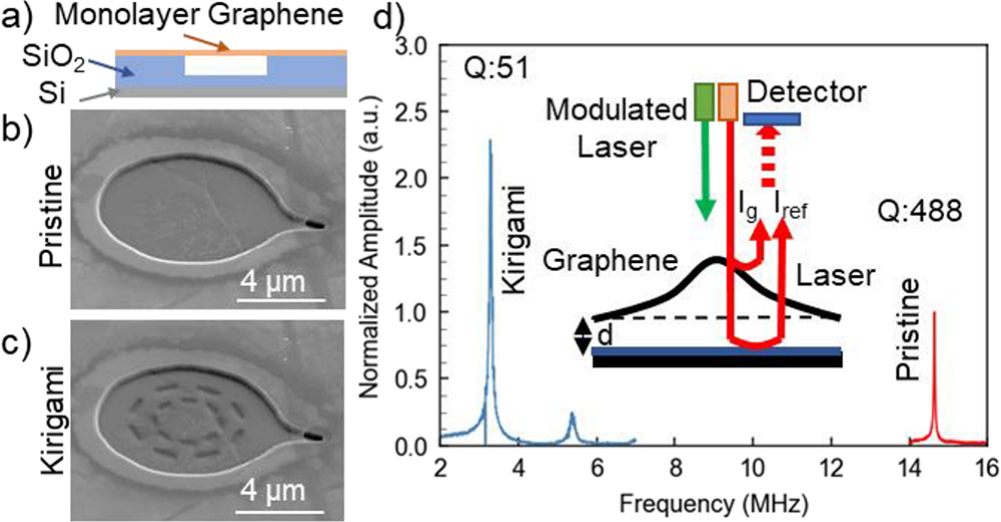
Optomechanical characterization of the resonant behaviors of the pristine and circular kirigami graphene. (a–c) Configuration and SEM images of the mechanical transducer setup for (b) pristine and (c) circular kirigami graphene. The circular kirigami pattern is formed on the suspended monolayer graphene membrane using a gallium ion beam. (d) Plot of the resonant peaks of pristine and kirigami graphene membranes. The measurement is conducted in the vacuum with a pressure around 10^–6^ Torr. The measured amplitudes are normalized to the amplitude of pristine graphene. The inset is the illustration of the measurement setup and mechanics. A modulated laser (green) is used to actuate the graphene membrane. A secondary probe laser is used for detection. The intensity of the interfered reflected signal from the graphene surface (*I*_g_) and substrate (*I*_ref_) is detected by a photodiode, which further resolves the resonance of the membranes.

Figure 2b and c shows the pristine and circular kirigami graphene membranes used for transduction characterization. After transferring graphene to the previously formed wells, a small hole is drilled in the graphene suspended above a short substrate channel to release any air trapped in the cavity and to maintain the same pressure across the membrane during actuation (Figure 2b). This “pressure relief” also significantly improves the yield for milling kirigami patterns on the suspended graphene (Figure 2c). Figure 2d shows the measured resonant behaviors of the pristine and circular kirigami graphene. Except where noted, all measurements are performed in a vacuum chamber with pressure less than 10^–6^ Torr to minimize the effect of air damping. Typically, the modulated driving laser power is set at 0.05 mW (Figure 2d inset). For the device shown in Figure 2, the resonant frequency of the pristine graphene membrane is 14.6 MHz. After modification with a circular kirigami pattern, the resonance is shifted to 3.3 MHz, a 77.4% reduction. The kirigami pattern also yields a displacement enhancement of 119%, and a reduction in Q from 488 to 51, a nearly 10× decrease. Hence, the kirigami modification meets all the three requirements for miniaturizing a wideband resonator: reduced resonant frequency, higher amplitude, and broader bandwidth. We find that pristine and circular cut kirigami graphene can survive many drive cycles without obvious fatigue or damage. In particular, we see no evidence of failure as might arise from theoretically proposed bond reconfiguration.^28^

We gain significant insight into these observations through modeling (see SI for technical details of the modeling program). Figure 3 shows the finite element simulation results for the fundamental mode displacement for a circular, pretensioned, suspended monolayer graphene membrane of diameter 8 μm driven by a uniformly distributed sinusoidal ac forcing function acting normally to its surface. Figure 3a–c shows maximum displacement snapshots respectively for pristine (uncut) graphene, graphene cut with a circular kirigami pattern, and graphene cut with a spiral kirigami pattern. Figure 3d shows simulated results for the fundamental mode frequency versus preapplied (isotropic) stress for pristine, circular kirigami cut, and spiral kirigami cut patterns. The square-root dependence of mode frequency versus stress in Figure 3d for the pristine membrane is expected. For a circular resonator under tension, the vibration mode reflects a standing wave formed from the superposition of traveling transverse waves in the circular membrane with a fixed boundary. The fundamental mode frequency *f* is given analytically by
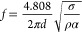
2where σ is the built-in stress on the membrane, ρ is its mass per unit, *d* is the diameter of the membrane, and α is a density multiplier.^15^ Importantly, relaxing built-in stress can dramatically shift down the resonant frequency of a suspended graphene membrane.

**Figure 3:**
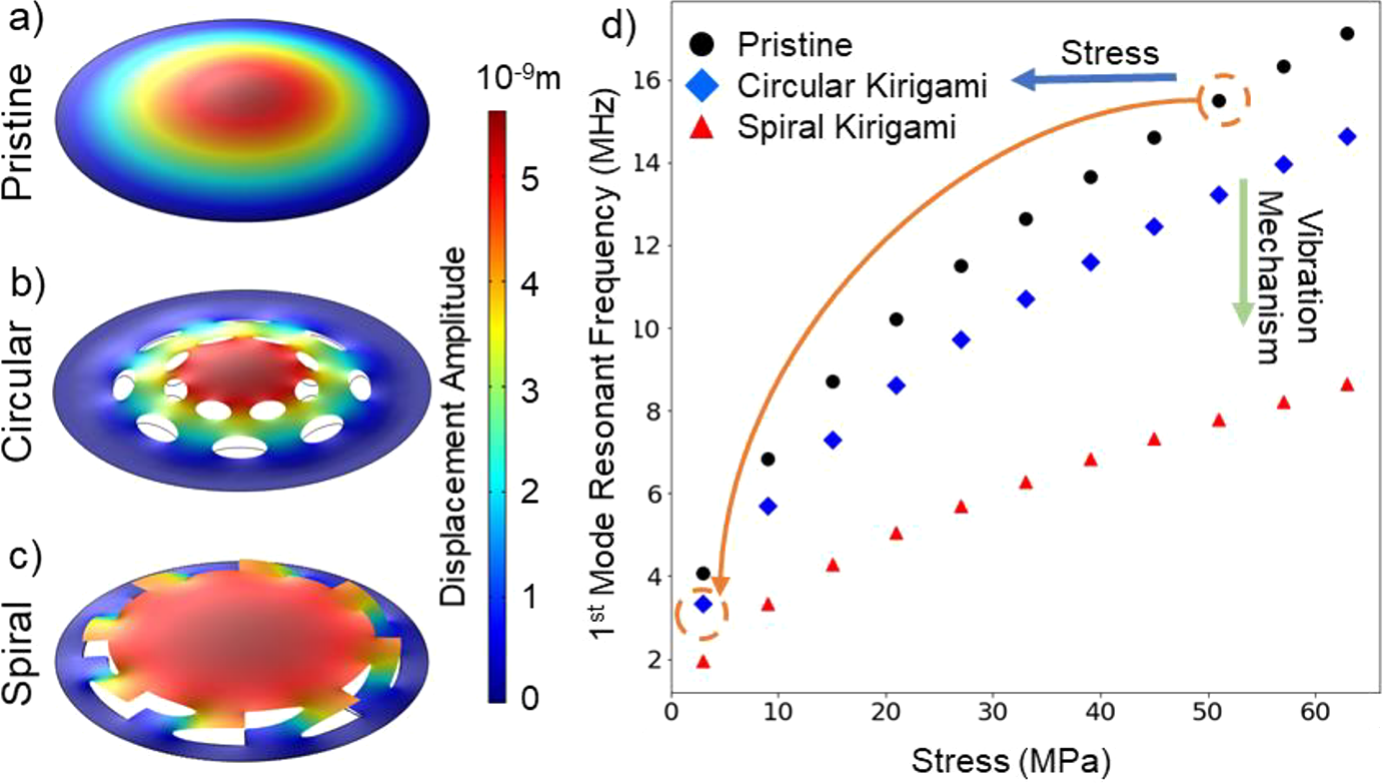
Simulation of the mechanical properties on the pristine and kirigami graphene membrane. (a–c) First and second resonant modes of pristine graphene, circular kirigami, and spiral kirigami. The black lines shown in panels b and c are the boundary of membrane before actuation. (d) Effect of stress on the resonant frequency on the three different membranes. The orange arrow identifies the transition of the graphene membrane after kirigami patterning.

From Figure 3, we learn that kirigami cuts have multiple beneficial effects. First, if a suspended graphene membrane is created with some built-in stress (which is inevitable using conventional fabrication methods), subsequent kirigami cuts can substantially relieve that stress and thereby lower the mode frequency. This effectively shifts the operating point leftward in Figure 3d. Second, and equally important, the kirigami cuts completely alter details of the vibration mode, including mode frequency and amplitude profile. Even for the same applied stress, the fundamental mode frequency is substantially lower than that predicted by eq 2 for the uncut membrane, for example by a factor of 2 for the spiral pattern. Kirigami cutting effectively softens the membrane and shifts the operating point downward in Figure 3d. In practice, both effects conspire to dramatically shift the mode frequency down upon kirigami cutting. For example, starting with a pristine graphene membrane prestressed to 50 MPa (upper-right dashed circle in Figure 3d), a circular kirigami cut in that membrane can reduce the stress to 2.5 MPa and further soften the membrane, such that the final resonance frequency is lowered from approximately 15 to 3.5 MHz (left-lower dashed circle in Figure 3d), a reduction by a factor of 4.3. The resonant frequencies could be further precisely engineered by tuning the parameters of the slots (see Figure S6 for details). We also note that kirigami cuts not only serve to reduce built-in stress but also tend to homogenize it, as evidenced by the elimination of stress ripples across the membrane upon cutting (Figure S7).

Alteration of the vibration mode profile upon kirigami cutting can be quite dramatic, as seen by comparing Figure 3a with Figure 3c. Indeed, the mode profile for the spiral kirigami cut resembles more that of a rigid circular plate suspended at its edges by very soft springs, rather than that of a vibrating drumhead. This has direct consequences for membrane displacement amplitude, at the geometrical center of the membrane *and* integrated over its surface area. The response motivates a description using a simple harmonic oscillator where the (undamped) response frequency is given by
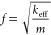
3where *k*_eff_ is the effective restoring spring constant of the combined soft flexures at the edges of the plate, and m is the mass of the plate. With sufficiently soft flexures (achieved, for example, via sufficiently thin and long flexures, or by overlapping or nesting the kirigami cuts^12,29,30^), *k*_eff_ can be made exceedingly small, leading to an ultralow resonance frequency for a given membrane diameter. Furthermore, the softer the spring constant, the greater the displacement amplitude for a given forcing function. As shown in Figure 3c, this enhanced amplitude can be *uniform* across the entire membrane, resulting in high energy transduction efficiency.

We now examine experimentally the influence of drive power on the membrane response, for pristine and circular kirigami-modified membranes. The diameters of the membranes are 8 μm. Figure 4 shows response amplitude versus drive laser power for 0.1, 0.01, 0.005, and 0.001 mW drive power. The resonant frequencies of both membranes shift upward with increased optical drive power. Higher optical drive power likely induces more tension (via graphene’s negative thermal expansion coefficient) in the thin film and causes the increase of the resonant frequency. This observation also aligns with the aforementioned relationship between the resonant frequency and stress (Figure 3d). In addition, the kirigami pattern leads to enhanced signal amplitude, that is, enhanced membrane displacement. The amplitude of all the measured signals is normalized to the pristine membrane actuated by the laser with a power of 0.1 mW (Figure 4). Compared to the pristine graphene membrane, the response amplitude for the kirigami modified membrane is increased by as much as a factor of 5 at a laser power of 0.1 mW. This confirms that kirigami modification allows not only a lowering of the resonance frequency and a broadening of the bandwidth but also can result in a dramatic enhancement of the displacement amplitude.

**Figure 4:**
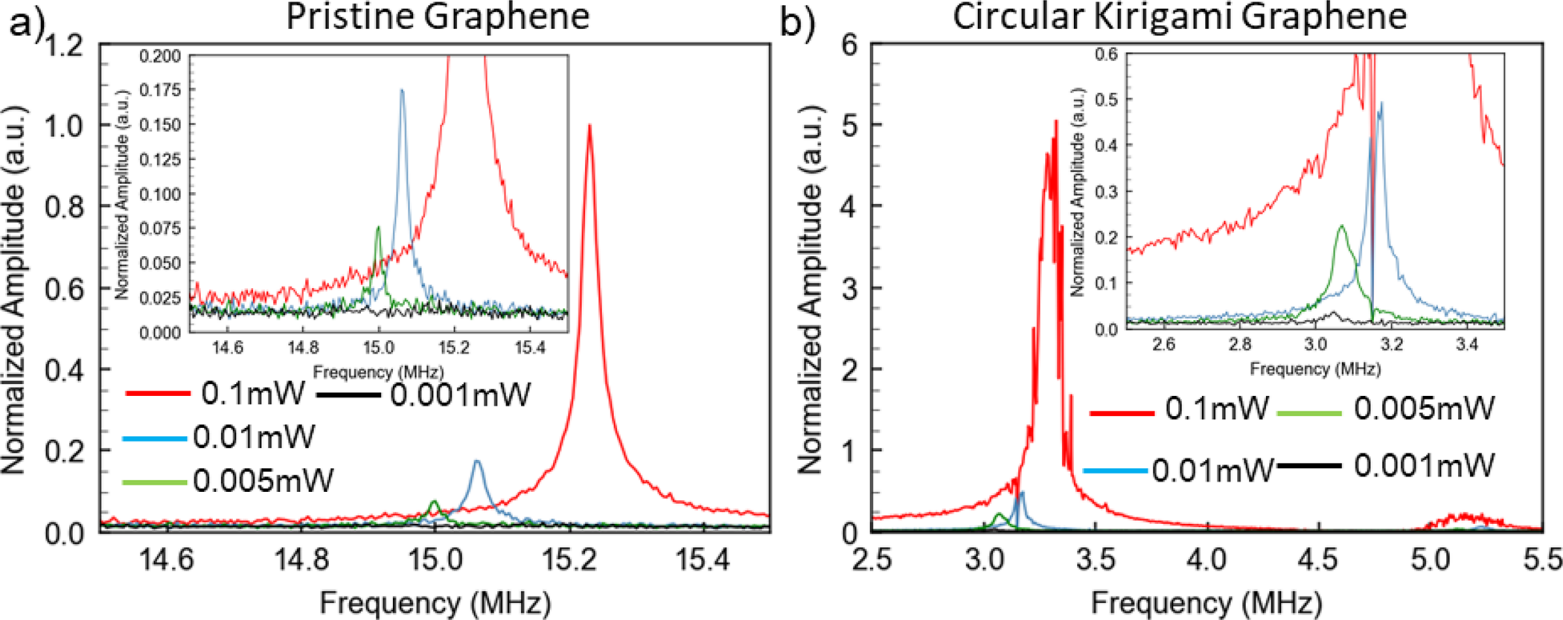
Comparison of the resonant modes of (a) pristine and b) circular kirigami graphene under laser with different actuation power, 0.1, 0.01, 0.005, and 0.001 mW. The membranes are 8 μm in size. The measurement is conducted in vacuum with a pressure around 10^–6^ Torr. The measured amplitudes are normalized to the amplitude of pristine graphene under the laser power of 0.1 mW. The inset is the zoomed-in images of the resonant peaks under laser irradiation with lower power. Enhanced displacement is observed on the circular graphene kirigami membrane.

Although our above presentation has been for transducers operated in vacuum, an interesting question is what benefit kirigami engineering might have for graphene transducers operated in a nonvacuum environment, for example in ambient air for ultrasonics or audio transduction. Because of the experimental constraints, we have not explored this “high pressure” regime. However, in a preliminary test in air at 0.7 Torr (Figure S9), we find for an 8 μm diameter circular cut membrane a change in bandwidth from 0.07 (measured in vacuum) to 0.12 MHz, a 71% increase. This suggests benefits for kirigami patterning in the finite pressure regime as well.

In conclusion, we have investigated kirigami modifications to suspended circular graphene membranes. Kirigami modification can both release built-in tension and change the vibration mechanism of a membrane, which in turn reduces the resonant frequency, decreases the *Q*, and increases the displacement amplitude. These trends are favorable for the miniaturization of wide-band thin-membrane transducers.

## Supporting Information Available

Sample fabrication, laser ablation system, TEM characterization of graphene kirigami after actuation, optical setup for device characterization, large displacement induced failure of characterization, finite element simulation setup, stress release during ion beam patterning, simulation of membrane displacement, and effect of pressure on membrane (PDF)
