# Supplementary material for: Kirigami Engineering of Suspended Graphene Transducers
Source: Nano Lett. 2022 Jun 27;22(13):5301–6. doi: 10.1021/acs.nanolett.2c01256 (PMC9284606; doi:10.1021/acs.nanolett.2c01256)
Supplement: Supplementary file 1 — nl2c01256_si_001.pdf [file nl2c01256_si_001.pdf]

Supporting Information

Kirigami Engineering of Suspended Graphene Transducers

Chunhui Dai[1,2,3†,] Yoonsoo Rho[4†], Khanh Pham[1], Brady McCormick[1], Brian W. Blankenship[4], Wenyu Zhao[1], Zuocheng Zhang[1], S. Matt Gilbert[1,2], Michael F. Crommie[1,2,3], Feng Wang[1,2,3], Costas P. Grigoropoulos[4], Alex Zettl[1,2,3*]

1. Department of Physics, University of California, Berkeley, CA 94720, USA.
2. Materials Sciences Division, Lawrence Berkeley National Laboratory, Berkeley, CA 94720, USA
3. Kavli Energy NanoSciences Institute at the University of California, Berkeley, CA 94720, USA.
4. Department of Mechanical Engineering, University of California, Berkeley, CA 94720
† These Authors contributed equally
*Corresponding Author

Table of Content:

1. Sample Fabrication ………..………………………………..…………………………2
2. Laser Ablation System……………………...……………………………..………4
3. TEM Characterization of Graphene Kirigami After Actuation……………..…………..5
4. Optical Setup for Device Characterization……………………………………..……6
5. Large Displacement Induced Failure of Characterization……………………..…….7
6. Finite Element Simulation Setup………………………………………………..……...9
7. Effect of the Design of the Kirigami Patterns……………………………………………10
8. Stress Release during Ion Beam Patterning…………………………………………...11
9. Simulation of Membrane Displacement…………………………………………………12
10. Effect of Pressure on Membrane ……………………………………………………...13

Sample Fabrication:

To prepare the graphene kirigami sample for TEM characterization, a monolayer commercial CVD graphene (GROLLTEX) is transferred on to a silicon nitride (Si_3N_4) transmission electron microscope (TEM) grid with an array of 10 μm holes through a wet transfer process (Figure S1a). For the transfer, the CVD graphene sample is spin-coated with a polymethyl methacrylate (PMMA) layer. The uncoated graphene on the backside of copper (Cu) foil is removed by oxygen plasma. Next, the sample is put in the sodium persulfate solution to etch the copper (Cu) substrate, leaving the PMMA/graphene floating on the surface of the solution. Then, it is taken to a DI water bath and transferred onto the TEM grid. After baking on a hot plate at 100 °C for 5 minutes, the sample is annealed in a furnace with a mixture of argon (Ar) and hydrogen (H_2) at 350 °C for 3 hours. Finally, a helium/gallium ion beam is used to create the kirigami patterns on the suspended graphene membrane (Figure S1b). The ion beam voltage is 25 kV.

To fabricate the graphene kirigami sample for transduction characterization, an array of wells is first defined on a silicon (Si) / silicon dioxide (SiO_2) wafer using photolithography and plasma etching. Photoresist S1818 is spun on top of the Si/SiO_2 wafer at 4000 rpm and the sample is baked at 115 °C for 1 minutes (Figure S1c). Then, an array of 8-μm holes is patterned on the photoresist using a direct-write photolithography system (MicroWriter ML3) (Figure S1d). After developing in MF319, SiO_2 on the uncovered hole areas is etched using a reactive ion etching process with a mixture of CHF_3 and O_2 (Figure S1e). The depth of the well is around 800nm. After the photoresist mask is removed using acetone (Figure S1f), a monolayer graphene is transferred above the well using the aforementioned wet transfer process (Figure

S1g). Next, helium/gallium ion beam is used to create the graphene kirigami patterns (Figure S1h).

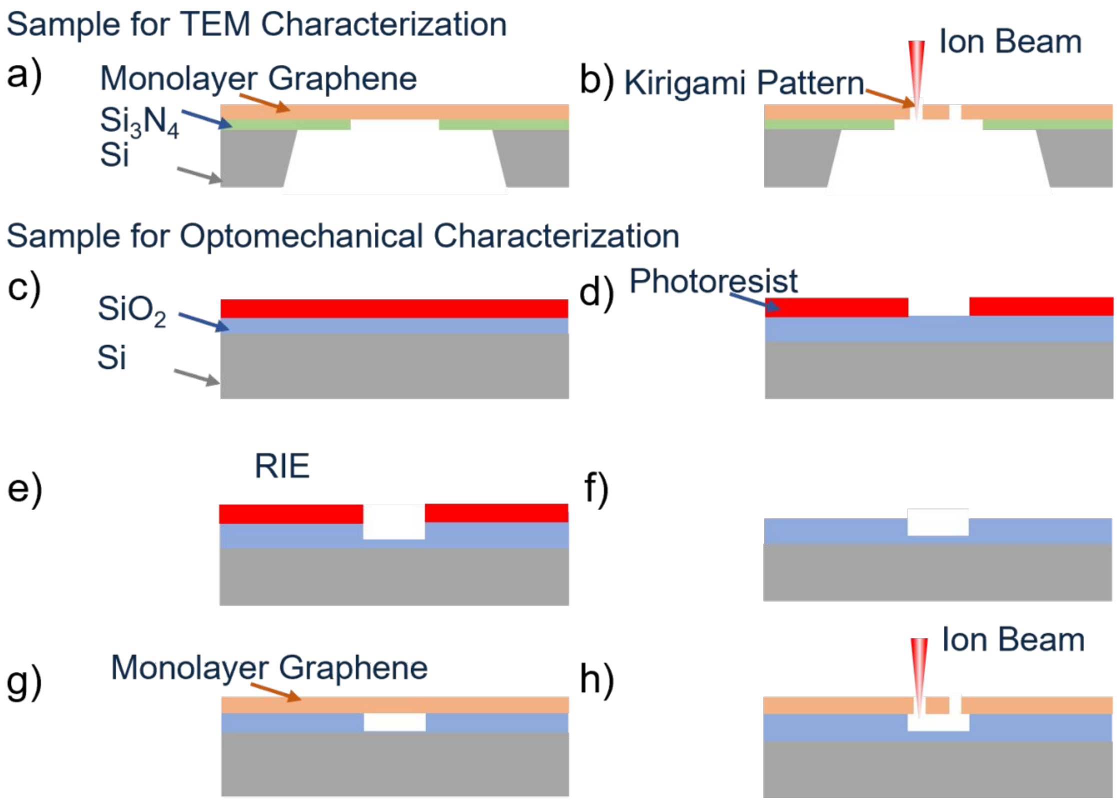

Figure S1，Fabrication processes of graphene kirigami samples prepared for a,b) TEM characterization and c-h) optomechanical measurement.

Laser Ablation System:

For the laser patterning process, we utilize an amplified femtosecond pulsed laser (Spitfire, Spectra-Physics, frequency~1 kHz, pulse duration~100 fs, pulse energy ~1 mJ). We adjust the laser energy by half-wave plate and polarized beam splitter down to ~1 μJ and focus the laser to the sample surface using either 50X or 10X objective lenses, resulting ~2 μm and 10 μm of spot sizes, respectively. We monitor the sample while patterning by introducing a dichroic mirror to reflect the processing beam while transmitting white light for imagining. For the patterning, the sample is placed on a motorized XY stage and scanned at ~5 mm/s.

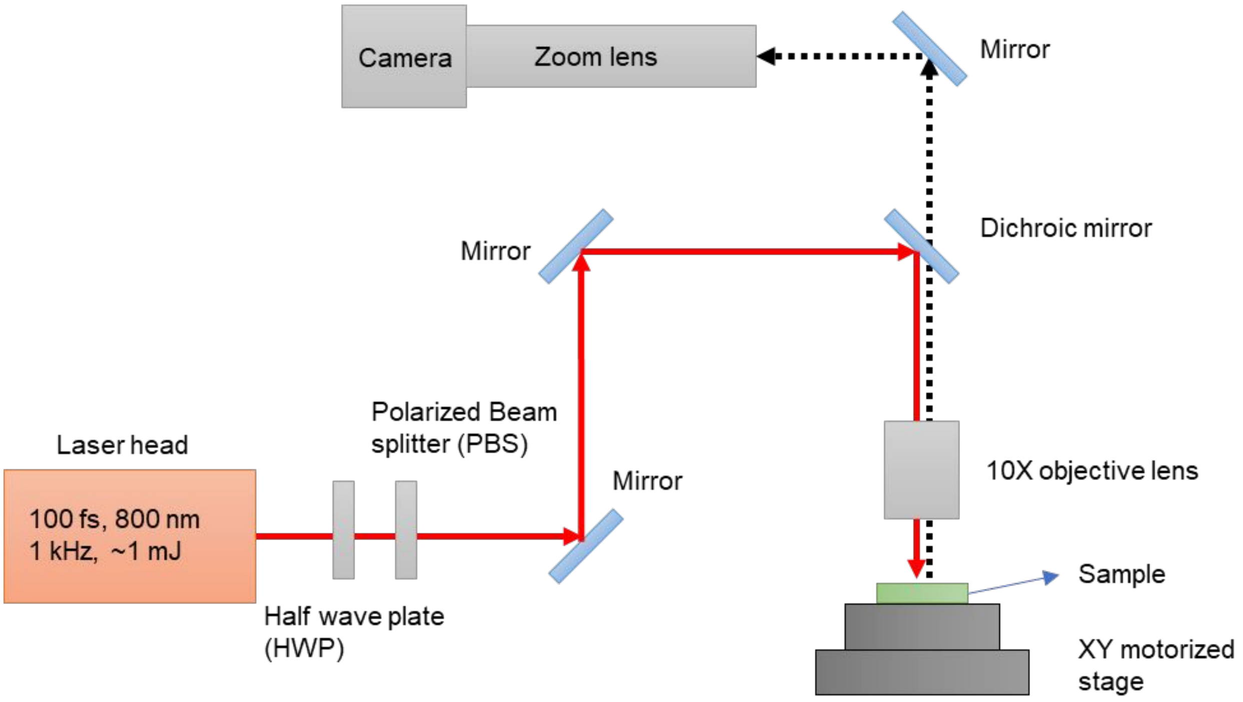

Figure S2, a) The morphology change of graphene kirigami patters are confirmed by TEM. b) TEM diffraction patterns show the preservation of the graphene crystal structures in the unirradiated area.

TEM Characterization of Graphene Kirigami After Actuation:

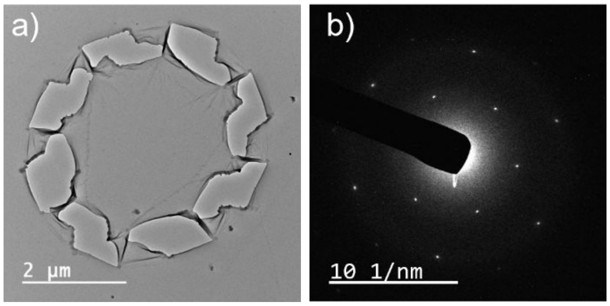

Figure S3, a) TEM image of a spiral-kirigami-cut membrane after sustained heavy actuation. The membrane has not catastrophically failed and still operates as a transducer, but the outside edges of cut corners have irreversibly folded, changing the local geometry. b) TEM diffraction patterns show the preservation of the graphene crystal structures in central suspended region. This demonstrates at neither irradiation from cutting, or sustained actuation, compromise the crystallinity of the relevant active portion of the membrane.

Optical Setup for Transduction Characterization:

For device characterization, we utilize two lasers including 532 nm CW laser for optically actuating the graphene membrane by optical pressure or thermal expansions and 633 nm He-Ne CW laser for optical probing to measure the reflectance. The intensity of 532 nm CW laser is modulated by acoustic optical modulator (AOM) whose frequency is controlled by function generator. Then, the modulated 532 nm laser beam is reflected by dichroic mirror for in-situ monitoring of the sample, goes through the polarized beam splitter (PBS), and finally focuses on the sample by 20 X objective lens. Meanwhile, the 633 nm probing laser beam are combined with 532 nm laser beam by PBS and passes through λ/4 plate before focusing to the sample. Next, the reflected 633 nm laser beam passes through λ/4 plate again, and the polarization of the laser beam rotates 900, allowing it passes through the PBS. Then, the reflected 633 nm probing beam goes to photodiode for detection. The signal from photodiode is demodulated by lock-in amplifier at the frequency provided by the function generation, which is the same frequency of the actuating 532 nm laser beam.

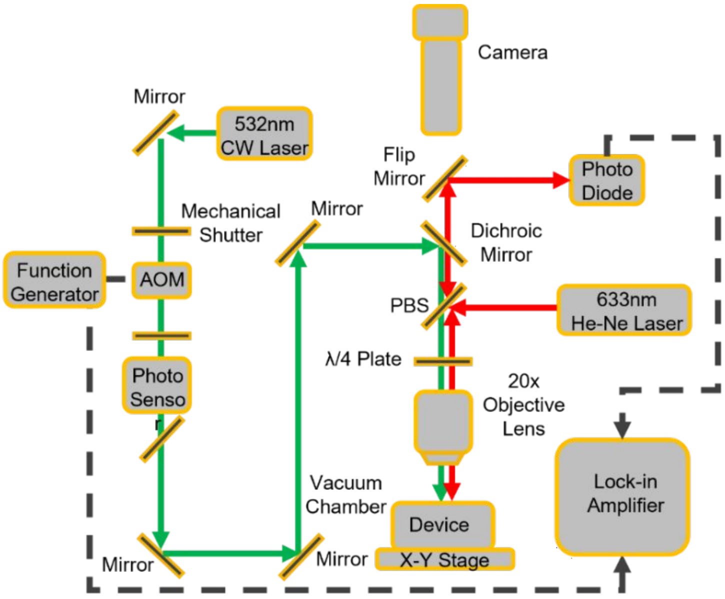

Figure S4, Schematics of optical setup for optomechanical characterization.

Large Displacement Induced Failure of Characterization:

As shown in Fig. 2D, the intensity of the interfered signals from the surface of the graphene membrane and the Si substrate is:

$$I(t) = 2\sqrt{I_g I_{ref}}\cos(\emptyset(t) + \emptyset_0) \quad (1)$$

where, I_g is the intensity of signal reflected from graphene surface, I_{ref} is the intensity of signal reflected from the silicon substrate, $\emptyset_0 = \frac{4\pi}{\lambda}d$ is the phase induced by the initial distance (d) between the suspended graphene and Si substrate, $\emptyset(t) = \frac{4\pi}{\lambda}x(t)$ is the phase induced by the displacement of the graphene membrane (x(t)), λ is the wavelength of the detection laser light. As the membrane experiences oscillatory motion, the displacement of the graphene membrane $x(t) = x_0\cos(\omega_0 t)$, where ω_0 is the resonant frequency. When the displacement of graphene is much smaller than the laser wavelength, equation (1) could be expressed as:

$$I(t) = 2\sqrt{I_g I_{ref}}(\ cos\emptyset_0 - \frac{4\pi}{\lambda}x_0\cos(\omega_0 t)sin\emptyset_0) \quad (2)$$

The intensity of the reflected signal is proportional to the displacement amplitude of the graphene membrane (x_0) at the oscillation frequency (ω_0) of the driving force. By feeding this signal together with the reference signal from the function generator into the lock-in amplifier (Figure S4), the intensity and phase of the vibrating graphene membrane can be directly extracted. However, this is not valid once the displacement of the graphene membrane is comparable or larger than the laser wavelength. In this situation, the intensity of the reflected signal is:

$$I(t) = 2\sqrt{I_g I_{ref}}\{\cos[\frac{4\pi}{\lambda}x_0\cos(\omega_0 t)]cos\emptyset_0 - \sin[\frac{4\pi}{\lambda}x_0\cos(\omega_0 t)]sin\emptyset_0\} \quad (3)$$

It is not proportional to the displacement amplitude of the graphene membrane (x_0), leading to the failure of detection. The spiral kirigami patterns have large displacement, which can easily

surpass the laser wavelength. As a result, the detected signal can no longer resolve the real amplitude of the resonating graphene membrane but a group of noisy signals around the actual resonant peak (Figure S5). Therefore, to maintain the small displacement limit, an 8-um graphene membrane with circular kirigami patterns is used. (Figure 2c).

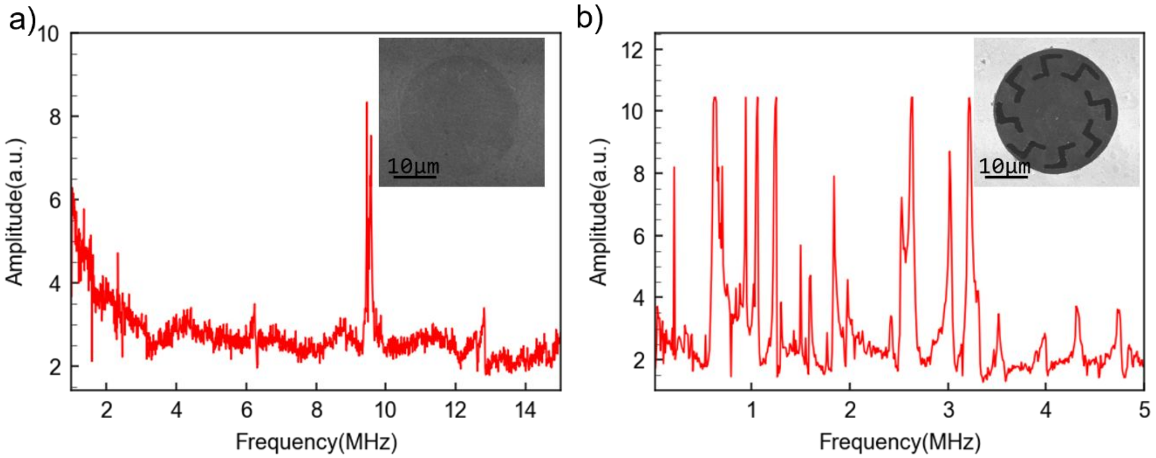

Figure S5，Optomechanical characterization of the resonant behaviors on a) pristine graphene and b) spiral graphene kirigami. b) The large displacement on spiral kirigami is beyond the detection limit of a Fabry Perot interferometry, leading to the noisy spectrum.

Finite Element Simulation Setup:

COMSOL Simulation Setup: The mechanical properties of the pristine and krigami graphene membrane were simulated using COMSOL Multiphysics. Standard graphene properties are used in the simulation, which has a density of 2.2 g/cm^3 and a Young's modulus of 1 TPa. The boundary conditions used are locking the outer edges (r(x,y) = 0 for (x,y) on the outer edges of the membrane).

Effect of the Design of the Kirigami Patterns:

The resonant modes and frequencies could be further tuned by varying the parameters of the kirigami patterns. As shown in Fig. S6, by increasing the length of the inner slots, the overlapped length between the inner and outer slots increases, which leads to a further decrease of the resonant frequency. This is due to the combined effects of further release of the built-in stress and modification of the resonant modes. Other parameters, such as number and distance of cuts, should also have the similar capability to further modify the resonance of the membrane towards the desired behavior.

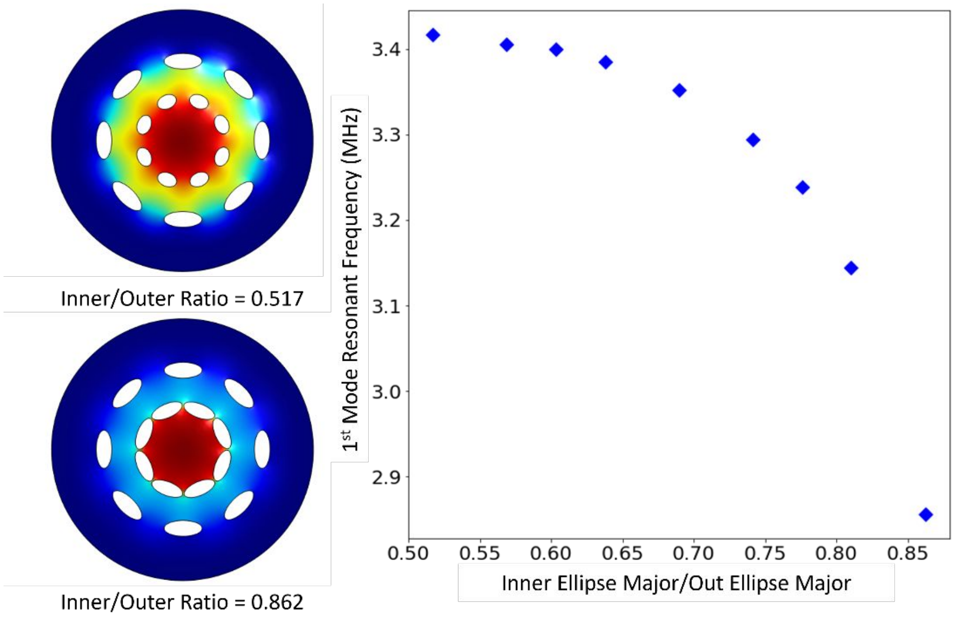

Figure S6, Finite element simulation showing the effect of different designs of circular kirigami graphene on the 1st mode resonant frequency. The higher ratio of the inner to outer ellipse major contributes to lower resonant frequency.

Stress Release during Ion Beam Patterning:

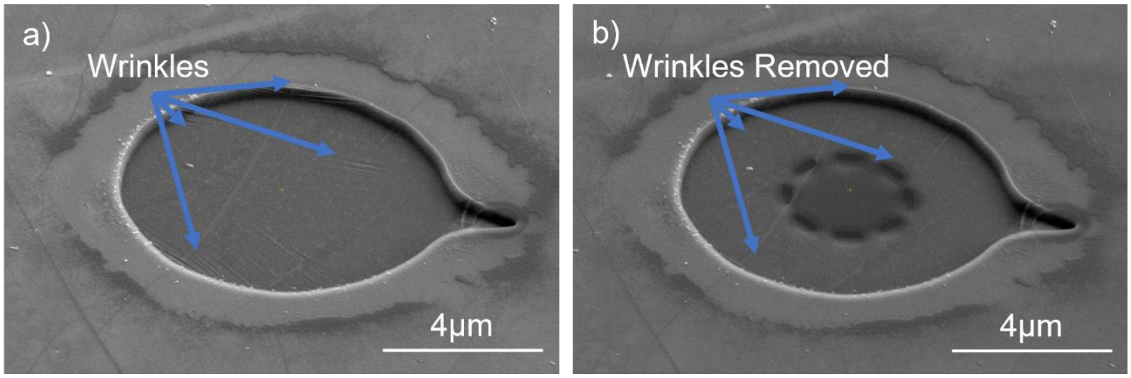

Figure S7, SEM images confirm the built-in stress is released after gallium ion beam milling.

Simulation of Membrane Displacement:

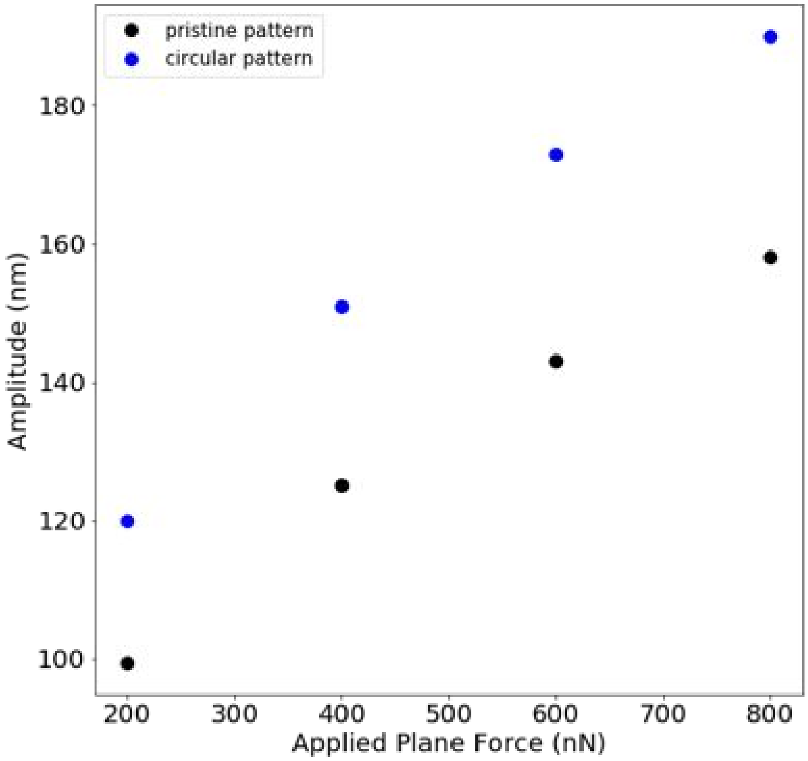

Figure S8，Simulation of the displacement of the pristine and circular graphene kirigami membrane with a diameter of 8μm under different loading forces. Larger displacements are always achieved on the kirigami membrane, which confirms the decrease on spring constant due to kirigmai pattern.

Effect of Pressure on Membrane:

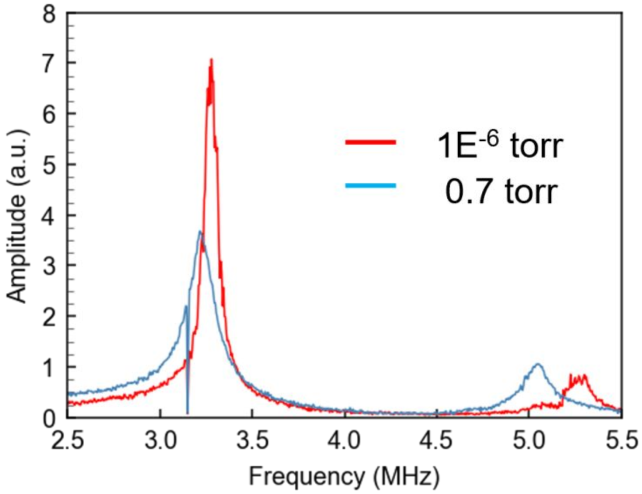

Figure S9，The effect of pressure on the resonant behavior of a monolayer circular graphene kirigami resonator with a diameter of 8μm.
